# Kidney stones and moderate proteinuria as the rare manifestations of Gitelman syndrome

**DOI:** 10.1186/s12882-020-02211-y

**Published:** 2021-01-07

**Authors:** Qi Chen, Xiaoyi Wang, Jingjing Min, Lin Wang, Lijun Mou

**Affiliations:** 1 Department of Nephrology, The First People’s Hospital of Huzhou, 313000 Huzhou, Zhejiang People’s Republic of China; 2 Department of Neurology, The First People’s Hospital of Huzhou, 313000 Huzhou, Zhejiang People’s Republic of China; 3 Guangzhou Kingmed Diagnostic Laboratory Ltd, 510320, Guangzhou, Guangzhou, Guangdong People’s Republic of China; 4 Division of Nephrology, Second Affiliated Hospital of Zhejiang University School of Medicine, No.88, Jiefang Road, Shangcheng District, Hangzhou, Zhejiang 310009 People’s Republic of China

**Keywords:** Gitelman syndrome, Case report, Diabetic nephropathy, Kidney stones

## Abstract

**Background:**

Gitelman syndrome (GS) is an autosomal recessive inherited salt-losing tubulopathy (SLT). Here, we describe, for the first time, a case of GS without Gitelman-like features and with concomitant kidney stones, cysts and diabetic nephropathy (DN).

**Case presentation:**

We described a male patient had a 19-year history of recurrent fatigue. From childhood, he had polydipsia and polyuria, paroxysmal tetany and palpitation. Serum biochemistry revealed chronic hypokalemia, metabolic alkalosis, normomagnesemia, mildly elevated Cr. Concomitant 24 h urine collection showed inappropriate renal potassium wasting, borderline hypercalciuria, moderate proteinuria consisting of major glomerular. Ultrasound of urinary tract showed bilateral and multiple kidney stones and cysts. Whole exome sequencing (WES) identified compound heterozygous mutations of *SLC12A3*. The unusual association of SLTs and glomerular proteinuria prompted us to perform a renal biopsy. Renal pathology showed renal involvement consistent with GS and early stage of diabetic nephropathy (DN). After treatment with KCl, magnesium oxide, perindopril and acarbose, the patient had been cured. The fatigue didn’t relapse.

**Conclusion:**

GS had high variability of phenotype, GS may have no Gitelman-like features, kidney stones are not the exclusion criteria of GS. Renal biopsy should be warranted for GS patients with moderate to massive glomerular proteinuria.

## Background

Gitelman syndrome (GS; OMIM 263800) is an autosomal recessive inherited salt-losing tubulopathy (SLT) characterized by hypokalemic metabolic alkalosis, hypomagnesemia, and hypocalciuria. GS is caused by biallelic inactivating mutations in the *SLC12A3* gene encoding the thiazide-sensitive sodium-chloride co-transporter (NCCT) in the distal convoluted tubule (DCT) [1, 2]. Previously, hypomagnesemia (< 0.7 mmol/L) with in appropriaterenal magnesium wasting and hypocalciuria were regarded as diagnostic criteria for GS. On the contrast, nephrolithiasis was considered as the one of the features against a diagnosis of GS due to hypocalciuria [3]. Additionally, SLTs including GS are not classically associated with proteinuria, especially glomerular proteinuria [4]. To date, GS without Gitelman-like features and with concomitant nephrolithiasis and glomerular proteinuria hasn’t been reported.

## Case presentation

**Fig. 1:**
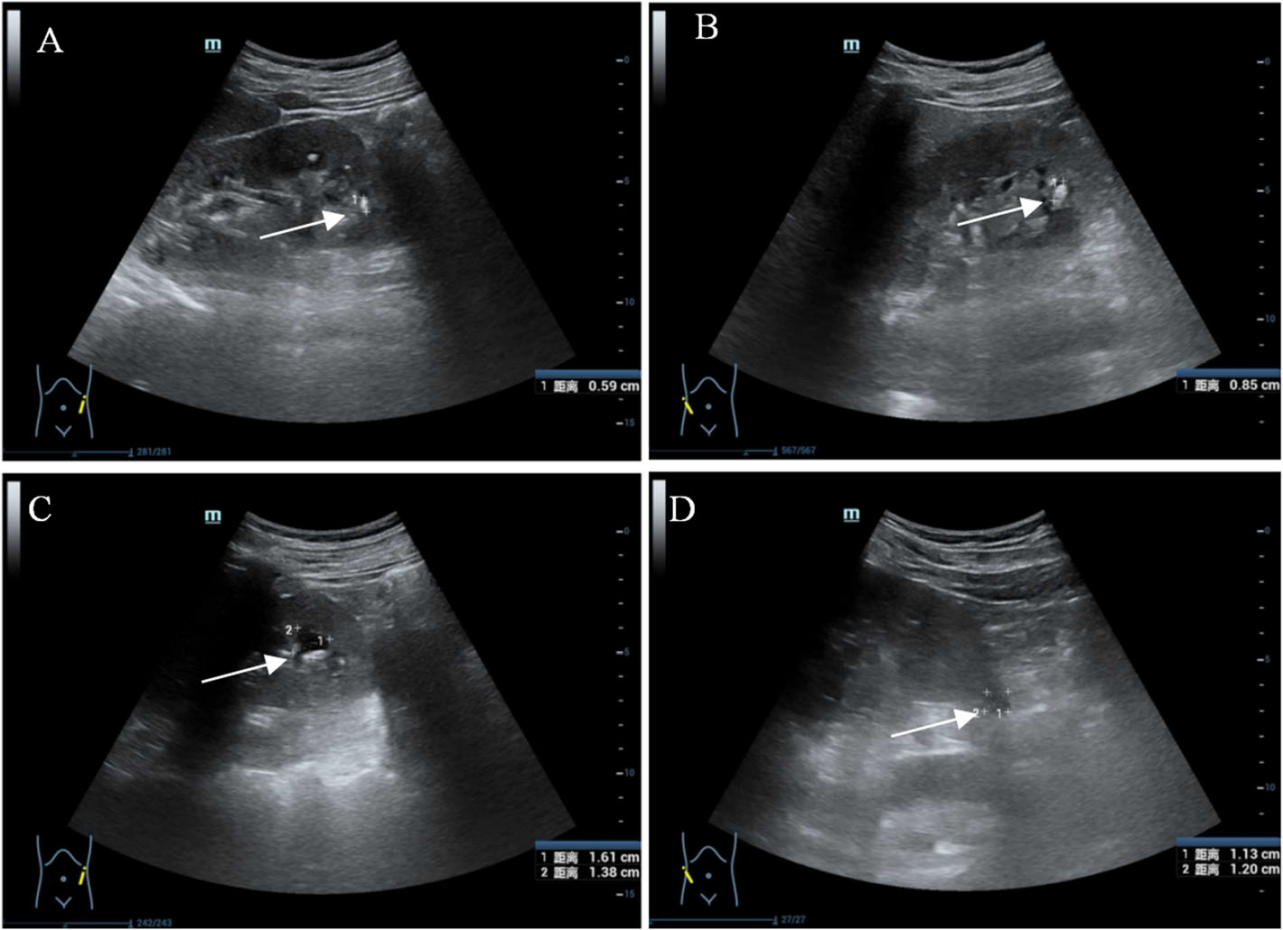
Ultrasound imaging of the urinary tract. **a** multiple kidney stones in the left kidney (arrow). **b** multiple kidney stones in the right kidney (arrow). **c** multiple kidney cysts in the left kidney (arrow). **d** multiple kidney cysts in the right kidney (arrow)

A 40-year-old, malepatient, was admitted to the hospital with a 19-year history of recurrent fatigue was hospitalized in The First People's Hospital of Huzhou on September 27, 2019.Generalized fatigue and dyspnea developed following upper respiratory infection 19 years ago, laboratory test revealed profound hypokalemia (serum potassium1.8 mmol/L), he was intubated and mechanic ventilation was applied due to respiratory failure resulting from respiratory muscle weakness. After intravenous potassium chloride (KCl) was administered, serum potassium was normalized, the fatigue and dyspnea was subsequently completely resolved. After he was discharged, he wasn’t on KCl supplement though generalized fatigue relapsed occasionally. Fatigue resolved by potassium-rich food intake. From 10 years ago, fatigue failed to be resolved by potassium-rich food intake. Therefore, he was on potassium supplement (KCl 2 ~ 3 g daily). Ten years ago, mild proteinuria developed, the amount of 24 h protein excretion was about 2 g, increased postprandial glucose level was revealed 2 years ago, but he did not take any glucose-lowering drug. From childhood, he had polydipsia and polyuria, paroxysmal tetany and palpitation, but had no salt craving, constipation, physical and mental retardation. His parents were not consanguineous, he had a younger sister and a son. His relatives are all healthy. Physical examination revealed a sitting blood pressure of 118/83 mmHg. His height was 170 cm, body weight 59.3 Kg, BMI 20.52 kg/m^2^.Serum biochemistry showed hypokalemia, hypochloridemia, normomagnesemia, mildly elevated creatinine (Cr). Concomitant 24 h urine collection showed inappropriate renal potassium wasting, borderline hypercalciuria, moderate proteinuria (2793 mg), and high oxalic acid and low citric acid. Arterial blood gas analysis revealed decompensated metabolic alkalosis. Urinalysis showed elevated microalbuminuria which indicated glomerular injury and elevated β2- microglobulinuria which indicated proximal tubular injury. Fasting blood glucose level was 7.33 mmol/L, the glucose level at 2nd hour of Oral glucose tolerance test (OGTT) was 11.27 mmol/L, both glucose levels confirmed the diagnosis of type2 diabetes mellitus (DM) (Table 1). Ultrasound of urinary tract showed bilateral and multiple kidney stones (Fig. 1a, b) and cysts (Fig. 1c, d).

**Table 1:** Laboratory investigations

Examination item	Test value	Reference value
Serum biochemistry		
Potassium (mmol/L)	2.0	3.50–5.30
Sodium (mmol/L)	139.0	137.0–147.0
Chloride (mmol/L)	98.0	99.0–110.0
Calcium (mmol/L)	2.15	2.08–2.60
Magnesium (mmol/L)	0.92	0.70–1.10
Phosphate (mmol/L)	0.86	0.85–1.51
Creatinine (μmol/L)	102.0	57.0–97.0
Uric acid (μmol/L)	438	208–428
Arterial blood gas analysis		
PH value	7.49	7.35–7.45
PaO_2_ (mmHg)	102.1	83.0–108.0
PaCO_2_ (mmHg)	37.0	35.0–45.0
HCO3^−^(mmol/L)	28	22–26
Base excess (mmol/L)	5.0	−3.0-3.0
Urine analysis		
Urine specific gravity	1.015	1.003–1.030
PH value	8.0	4.5–8.0
Albumin to creatinine ratio (mg/g.Cr)	1133.33	< 25
α1- microglobulin to creatinine ratio (mg/g.Cr)	44.6	< 15
24-h Urine tests		
Potassium (mmol/24 h)	253.10	25.00–100.00
Calcium (mmol/24 h)	5.34	0.25–7.5
Creatinine (mg/24 h)	1250.2	700.0–2300.0
Uric acid (mmol/24 h)	4666.0	1488.0–4463.0
Oxalic acid (mg/24 h)	93.05	20–40
Citric acid (mg/24 h)	80.18	> 450
Urinay calcium/creatinine (mmol/mmol)	0.48	
Renin-angiotensin-aldosterone system (upright)		
Plasma renin (μIU/ml)	> 500	4.4–46.1
Plasma aldosterone (pg / ml)	688	30–353
Glycosylated hemoglobin A1C (%)	6.0	4.0–6.0
OGTT tests		
Fasting blood glucose (mmol/L)	7.33	3.89–6.11
Postprandial blood glucose (2 h) (mmol/L)	11.27	3.89–7.78

**Fig. 2:**
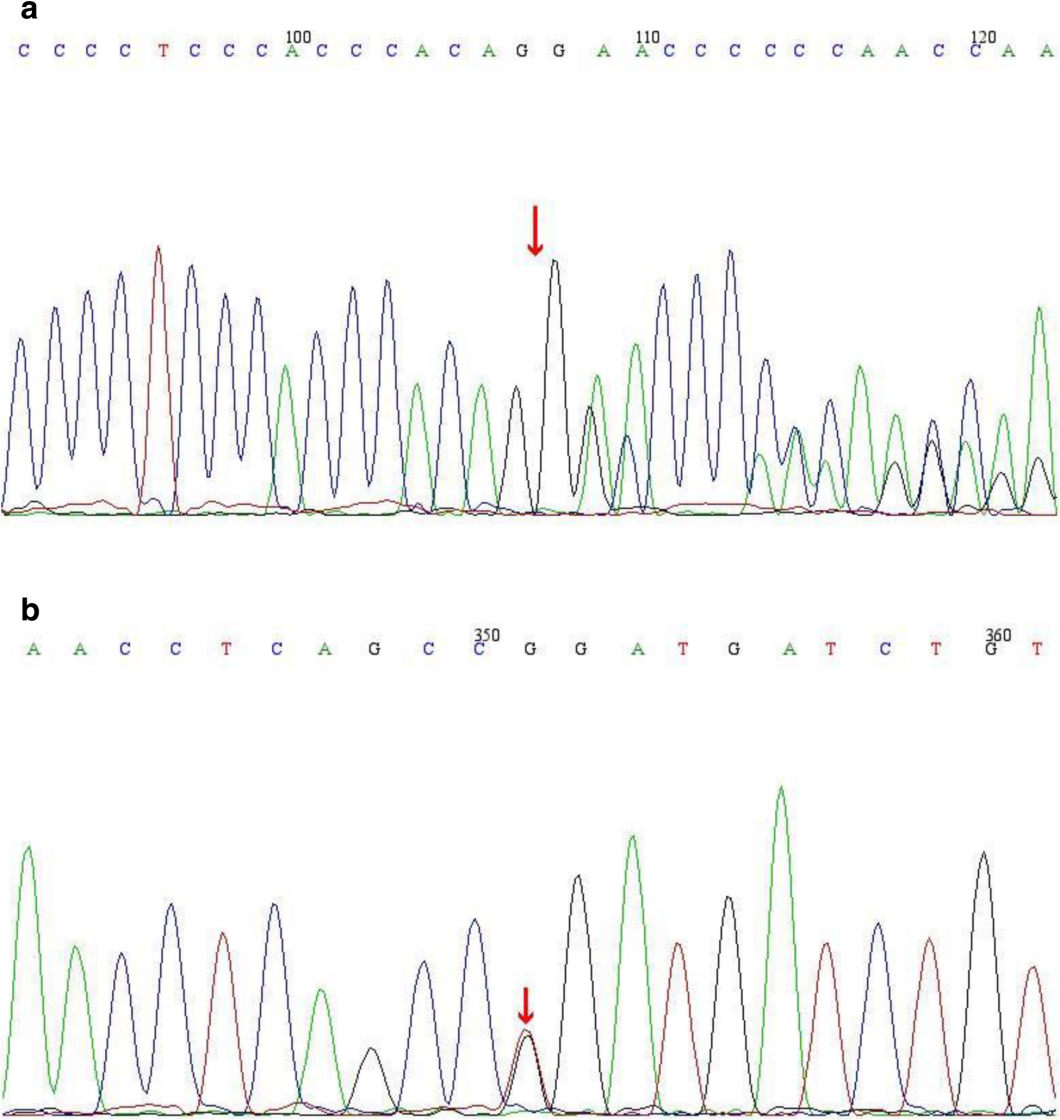
**a**. The patient and his father carried a c.2038-1delG (arrow) heterozygous mutation in *SLC12A3* gene, and his mother had no mutation in*SLC12A3* gene at this site. **b**. The patient and his mother’s carried a c.2012 T > G (arrow) (p.Leu671Arg) heterozygous mutationin*SLC12A3* gene, and his father had no mutation in *SLC12A3* gene at this site

Chronic hypokalemia resulting from urine potassium wasting, metabolic alkalosis, normal blood pressure despite elevated renin and aldosterone indicated SLTs. Therefore, whole exome sequencing (WES) was performed to identify the exact type of SLTs. Genomic DNA was extracted from blood peripheral leukocytes. Paired-end sequencing libraries then were prepared using a DNA sample prep reagent set 1 (NEBNext). The amplified DNA was captured use GenCap WES capture kit. The enrichment libraries were sequenced on IlluminaHiSeq X ten sequencer for paired read 150 bp.After sequencing, the bioinformatics analysis was performed. After quality control, the clean reads were mapped to the UCSC hg19 human reference genome using BWA. Variants were filtered using GATK VariantFiltration and further annotated by ANNOVAR and associated with multiple databases. The potential pathogenic mutations were selected. Candidate mutations were confirmed by Sanger sequencing. The mutations of family members were confirmed by the same procedure. WES revealed two heterozygous mutations in *SLC12A3* gene: one splicing mutation (c.2038-1delG), potentially leading to loss of function of the gene, one missense mutation (c.2012 T > G) resulting in arginine substitution for leucine at codon 671 (p.Leu671Arg). According to the American College of Medical Genetics (ACMG) mutation guidelines, the splicing mutation (c.2038-1delG) was classified as “pathogenic” and the missense mutation (c.2012 T > G) was classified as “likely pathogenic”.p.Leu671Arg was a novel mutation. His father carries a heterozygous mutation of c.2038-1delG (Fig. 2a), his mother carried a heterozygous mutation of p.Leu671Arg (Fig. 2b).

**Fig. 3:**
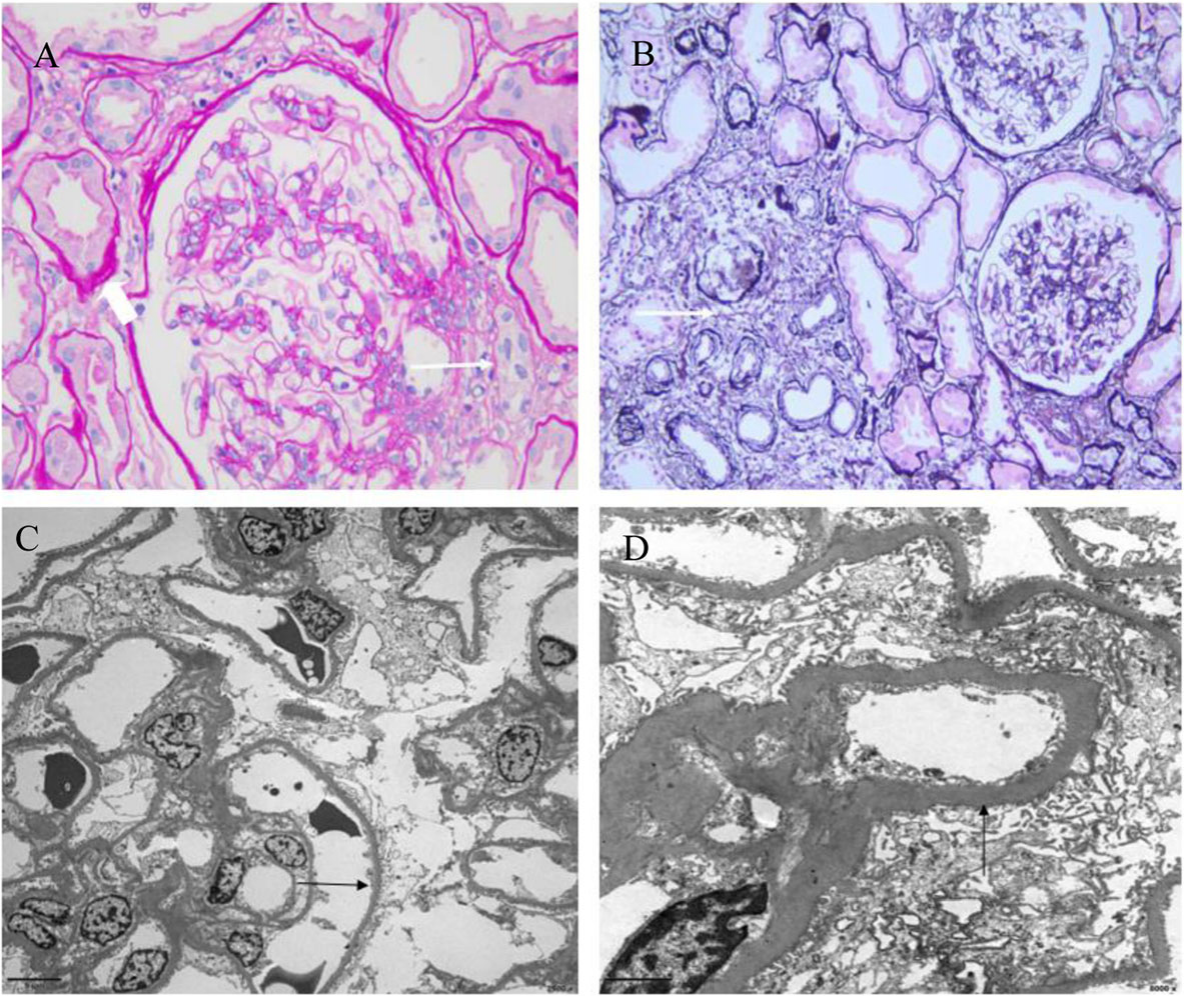
Renal pathology. **a** Light microscopic findingsof HE staining (magnification × 400) showedjuxtaglomerular apparatus proliferation (arrow) and granular and vacuolar degeneration of tubular epithelial cells (arrowhead). **b** Light microscopy findings of PASM staining (magnification × 200) showed multifocal atrophy of renal tubular, multifocal infiltration of inflammatory cells and fibrosis (arrow). **c** Electron microscopy (magnification × 2000) showed foot process effacement (50–75%) (arrow) and no electron dense deposits. **d** Electron microscopy (magnification × 8000) showed thickening GBM (744 nm)

The unusual association of SLTs and glomerular proteinuria prompted us to perform a renal biopsy. Light microscopy (LM) showed chronic tubulointerstitial nephritis and hypertrophy of the juxtaglomerular apparatus which are consistent with renal involvement resulting from GS; LM also revealed thickened basement membrane, mild mesangial cells and mesangial matrix proliferation, thickening arteriole wall and narrowing lumen. Immunofluorescence demonstrated no deposition of immunoglobulins or complements. Electron microscopy shows foot process effacement (50–75%) and thickening GBM with no electron dense deposits. The renal pathology also indicated early stage of diabetic nephropathy (DN) (Fig. 3).

Eventually, the diagnosis of GS without Gitelman-like features and with kidney stones, cysts and DN was established. KCl at the dose of 3 g three times daily, magnesium oxide 188 mg once daily and perindopril 4 mg once every night are administered to correct hypokalemia, acarbose100 mg three times to correct hyperglycemia. On Dec 20th, 2019, serum Cr was 104 μmol/L, serum potassium3.57 mmol/L and fasting blood glucose was 6.89 mmol/L.The fatigue didn’t relapse.

## Discussion and conclusion

We described a male patient had a 19-year history of recurrent fatigue. From childhood, he had polydipsia and polyuria, paroxysmal tetany and palpitation. Serum biochemistry revealed chronic hypokalemia, metabolic alkalosis, normomagnesemia, mildly elevated Cr. Concomitant 24 h urine collection showed inappropriate renal potassium wasting, borderline hypercalciuria, moderate proteinuria consisting of major glomerular. Ultrasound of urinary tract showed bilateral and multiple kidney stones and cysts. WES identified compound heterozygous mutations of *SLC12A3*.Renal pathology showed renal involvement consistent with GS and early stage of DN. To our best knowledge, this is the first case of GS without Gitelman-like features and with concomitant kidney stones, cysts and DN.

GS is an autosomal recessive, salt-losing tubulopathy. The prevalence of GS is approximately 1–10 per 40,000 persons in Western countries, and potentially higher in Asia [3]. Undoubtedly, identification of biallelic inactivating mutations in *SLC12A3* is the indispensable criteria for establishing a diagnosis of GS. In this case, WES revealed two heterozygous mutations in *SLC12A3* gene: one splicing mutation (c.2038-1delG), one missense mutation (c.2012 T > G) resulting in arginine substitution forleucine at codon 671 (p.Leu671Arg). p.Leu671Arg was a novel mutation. Therefore, it is indisputable that the patient was diagnosed with GS. Up to now, more than 400 *SLC12A3* gene mutation sites have been reported to be associated with GS, of which mutations in exons 1 and 10 are more common [5].

As is well known, GS was characterized by hypomagnesemia (serum magnesium < 0.7 mmol/L) and hypocalciuria (a spot urine calcium to creatinine ratio < 0.2 mmol/mmol), Kidney Disease: Improving Global Outcomes (KDIGO) guidance recommended both hypomagnesemia and hypocalciuria as diagnostic criteria for GS. On the contrast, nephrolithiasis was considered as the one of the features against a diagnosis of GS due to hypocalciuria [3]. However, a recent study from China showed that both hypomagnesemia and hypocalciuria were not sensitive enough to diagnose GS. The diagnostic sensitivity and specificity of hypomagnesemia for GS were 72.41 and 87.50%, respectively, the diagnostic sensitivity and specificity of hypocalciuriafor GS were 44.83 and 75.00%, respectively [6]. The present patient had normomagnesemia, borderline hypercalciuria, bilateral and multiple kidney stones and cysts which were all not consistent with typical GS. Eventually, gene sequencing identified the diagnosis of GS. Accordingly, GS had highly variable phenotype and gene sequencing was indispensable to diagnose correctly. Therefore, kidney stones and cysts are not exclusion criteria of GS. The mechanism of nephrolithiasis of the present case are unclear now, no relevant report was found in the Chinese and foreign literatures. In this case, the 24-h Urine tests showed high oxalic acid and low citric acid, indicating that the formation of kidney stones probably was a result of the increase in the urinary excretion rate of oxalic acid and the decrease in the urinary excretion rate of citric acid, which correlated with the increase in nephrolithiasis, on the other side, the polydipsia indicates insufficient capacity, which may further increase the risk of stone formation. The elevated β2- microglobulinuria which indicated proximal tubular injury, it may be associated with the increased reabsorption of sodium and calcium from proximal convoluted tubules. The connection between the kidney stones and cysts is unclear, the WES also excluded other 500 hereditary disease, including polycystic kidney.

SLTs including GS are not classically associated with proteinuria, especially glomerular proteinuria. However, in this case, the 24-h urinary protein excretion was 2793 mg, while most of the previously reported GS patients had normal or mildly elevated urinary protein [7, 8]. The findings of renal pathology were consistent with renal involvements of GS and early stage of DN. Therefore, for the GS patients with moderate to massive glomerular proteinuria, renal biopsy should to be warranted. Possible mechanisms between GS and glomerular proteinuria include angiotensin II or renin-induced podocyte lesions, as well as chronic hypokalaemia. One reason is that the chronic activation of the renin angiotensin-aldosterone pathway, leading to increased systemic and local levels of angiotensin-II and renin, may in turn cause podocyte lesions. Presently, the mechanism of GS with DN is still unclear, and some scholars have proposed that the dysfunction of NCC may be one of the main reasons for the risk of DN due to chronic hyperglycemia caused by insulin resistance in individuals with Type 2 Diabetes [9, 10]. In addition, Arg913Gln variation of *SLC12A3* gene is associated with DN in type 2 diabetes and GS [11]. A patient with GS and nephrotic syndrome was reported, the renal pathology revealed minimal lesions [7]. Maybe DN is coincidental to the GS, however, when glomerular proteinuria occurred in patients with GS, renal biopsy should be warranted in order to identify overlapping kidney diseases.

GS had high variability of phenotype, GS may have no Gitelman-like features, kidney stones are not the exclusion criteria of GS. Renal biopsy should be warranted for GS patients with moderate to massive glomerular proteinuria.

## Data Availability

Data regarding this study were obtained from clinical charts stored in the physician office records of The First People's Hospital of Huzhou therefore, cannot be shared. Any reasonable request to access the data must be approved before the data can be released.

## Abbreviations

GS: Gitelman syndrome
SLT: Salt-losing tubulopathy
NCCT: Thiazide-sensitive sodium-chloride co-transporter
DCT: Distal convoluted tubule
WES: Whole exome sequencing
DM: Diabetes mellitus
ACMG: American College of Medical Genetics
LM: Light microscopy
DN: Diabetic nephropathy
GBM: Glomerular basement membrane

## References

[1] Nakhoul F, Nakhoul N, Dorman E, Berger L, Skorecki K, Magen D (2012). Gitelman’s syndrome: a pathophysiological and clinical update. Endocrine.

[2] Gitelman Syndrome Collaborative Study G (2017). Expert consensus for the diagnosis and treatment of patients with Gitelman syndrome. Chinese J Internal Med.

[3] Blanchard A, Bockenhauer D, Bolignano D, Calo LA, Cosyns E, Devuyst O (2017). Gitelman syndrome: consensus and guidance from a kidney disease: improving global outcomes (KDIGO) controversies conference. Kidney Int.

[4] Demoulin N, Aydin S, Cosyns J-P, Dahan K, Cornet G, Auberger I (2014). Gitelman syndrome and glomerular proteinuria: a link between loss of sodium-chloride cotransporter and podocyte dysfunction?. Nephrol Dial Transplant.

[5] Takeuchi Y, Mishima E, Shima H, Akiyama Y, Suzuki C, Suzuki T (2015). Exonic mutations in the SLC12A3 gene cause exon skipping and premature termination in Gitelman syndrome. J Am Soc Nephrol.

[6] Peng X, Zhao B, Zhang L, Jiang L, Yuan T, Wang Y, et al. Hydrochlorothiazide test as a tool in the diagnosis of Gitelman syndrome in Chinese patients. Front Endocrinol. 2018;559. 10.3389/fendo.2018.00559 PMC6165878 30319542

[7] Chen Q, Wu Y, Zhao J, Jia Y, Wang W (2018). A case of hypokalemia and proteinuria with a new mutation in the SLC12A3 gene. BMC Nephrol.

[8] Fulchiero R, Seo-Mayer P (2019). Bartter syndrome and Gitelman syndrome. Pediatr Clin.

[9] Ren H, Qin L, Wang W, Ma J, Zhang W, Shen PY (2013). Abnormal glucose metabolism and insulin sensitivity in Chinese patients with Gitelman syndrome. Am J Nephrol.

[10] Yuan T, Jiang L, Chen C, Peng X, Nie M, Li X (2017). Glucose tolerance and insulin responsiveness in Gitelman syndrome patients. Endocrine Connect.

[11] De la Cruz-Cano E, CdC J-G, Morales-García V, Pineda-Pérez C, Tejas-Juárez JG, Rendón-Gandarilla FJ (2019). Arg913Gln variation of SLC12A3 gene is associated with diabetic nephropathy in type 2 diabetes and Gitelman syndrome: a systematic review. BMC Nephrol.

